# Supplementary material for: Preclinical PET Imaging of Tumor Cell Death following Therapy Using Gallium-68-Labeled C2Am
Source: Cancers (Basel). 2023 Mar 2;15(5):1564. doi: 10.3390/cancers15051564 (PMC10001225; doi:10.3390/cancers15051564)
Supplement: Supplementary file 1 [file cancers-15-01564-s001.zip › cancers-2100885-supplementary.pdf]

Supporting Information

Preclinical PET Imaging of Tumor Cell Death following Therapy using Gallium-68-Labeled C2Am

Flaviu Bulat [1,2*], Friederike Hesse [1], Bala Attili [1], Chandra Solanki [3], Iosif Mendichovszky [5], Franklin Aigbirhio [4], Finian J. Leeper [2], Kevin M. Brindle [1,6] and André A. Neves [1] .

[1]Cancer Research UK Cambridge Institute, University of Cambridge, United Kingdom
[2]Department of Chemistry, University of Cambridge, United Kingdom
[3]Addenbrooke's Hospital Radiopharmacy, Cambridge University Hospitals NHS Foundation Trust, United Kingdom
[4]Wolfson Brain Imaging Centre, University of Cambridge, United Kingdom
[5]Department of Biochemistry, University of Cambridge, United Kingdom
*Correspondence: Flaviu Bulat flaviu.bulat@cruk.cam.ac.uk; Kevin M. Brindle, kmb1001@cam.ac.uk;

Contents

Supporting Figures S1, S2, S3 and S4

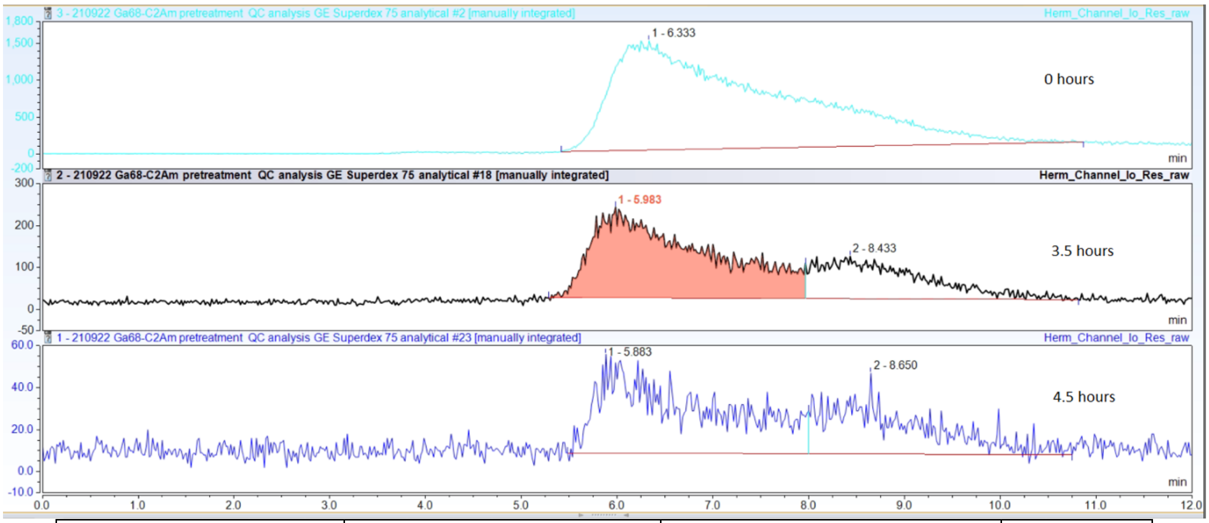

Time after EOS (hours)	Peak retention time (min)	^{68}Ga-C2Am relative area (%)	Area*min
0	6.333	>95%	2936
3.5	5.983	70	284
4.5	5.883	63	54

Supporting Figure S1 – Stability of ^{68}Ga-C2Am in PBS at 37 °C. Size exclusion radio HPLC chromatograms are show at times 0 (top), 3.5 h (middle) and 4.5 (lower). A single peak at ~6 min, corresponding to the ^{68}Ga-labelled protein.

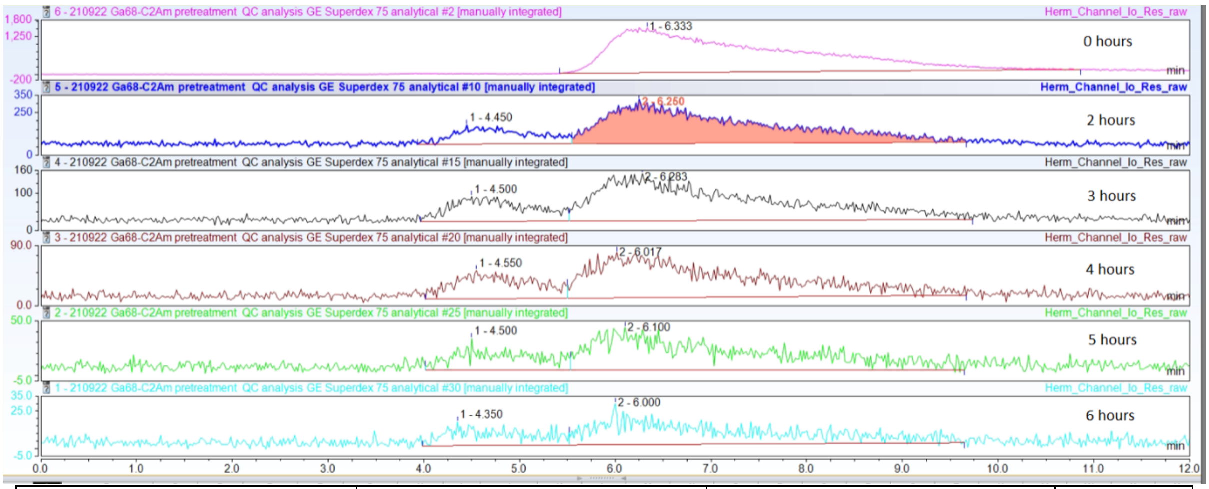

Time after EOS (hours)	Peak retention time (min)	^{68}Ga-C2Am relative area (%)	Area*min
0	6.333	>95	2936
2	6.25	82	417
3	6.283	79	224
4	6.017	77	118
5	6.1	79	66
6	6	75	33

Supporting Figure S2 – Stability of ^{68}Ga-C2Am in foetal bovine serum (FBS) at 37 °C. Size exclusion radio HPLC chromatograms are show at times 0, 2, 3, 4, 5 and 6 h. A single peak at ~6 min, corresponding to the ^{68}Ga-labelled protein, is visible at time 0. After 2 h, a second peak is visible at ~4.5 min, with an area ca. 20% of the major peak,

and corresponding to a species with greater molecular weight. The latter corresponds to a non-covalent adduct of ^{68}Ga-C2Am and serum albumin.

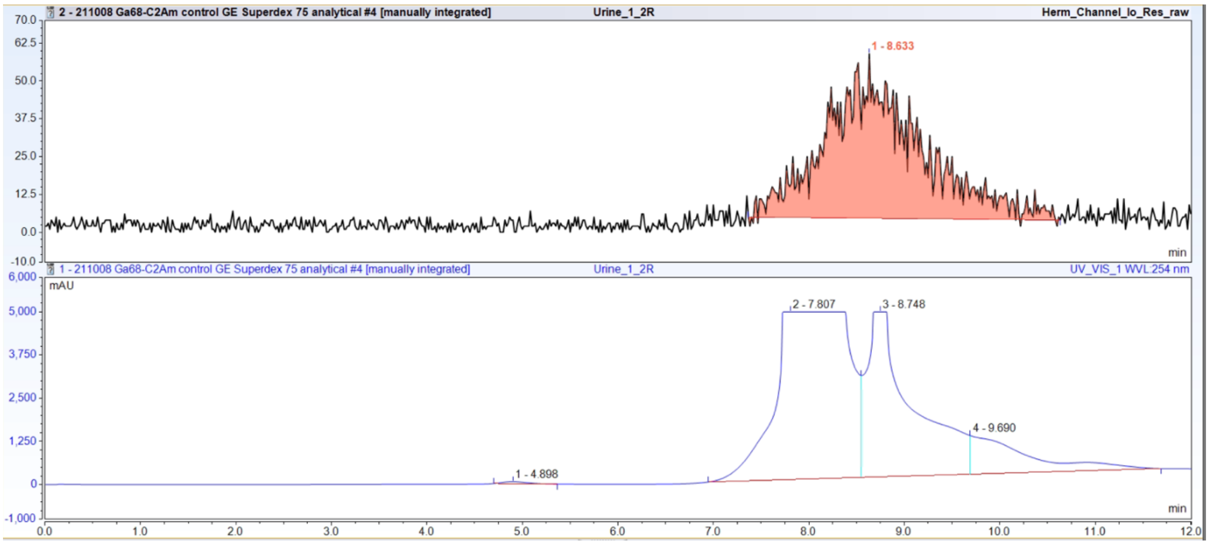

Supporting Figure S3 – Metabolites of ^{68}Ga-C2Am present in urine, 15 min after IV administration. Size exclusion radio HPLC (top) and UV (lower) chromatograms of mouse urine, showing a single peak at ~ 8.6 min, corresponding to small molecular weight (<3 kDa) metabolites of ^{68}Ga-labelled protein.

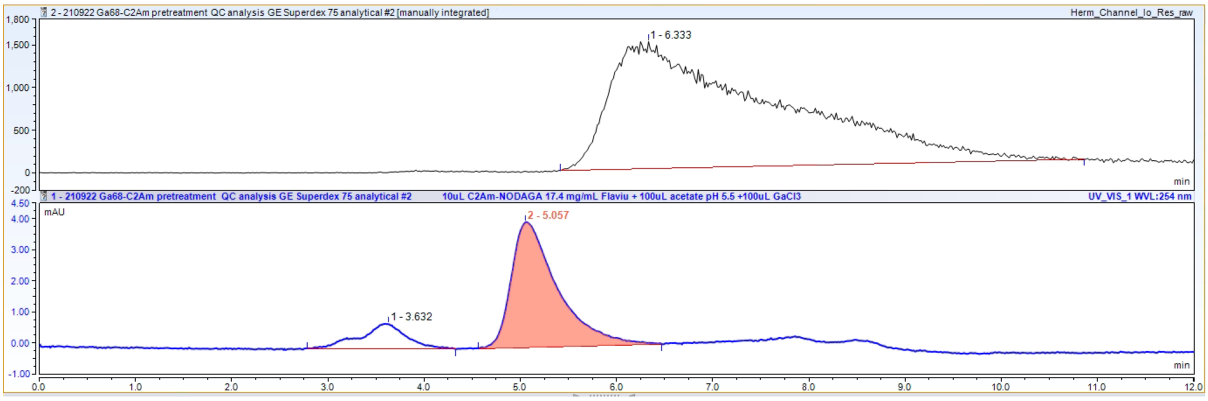

Supporting Figure S4 – Radiolabeling of C2Am using gallium-68. Size exclusion radio HPLC (top) and UV (lower) chromatograms, following radiolabeling of the protein. A single radiolabeled species (top) is detected at ~6.3 min corresponding to ^{68}Ga-C2Am. A second peak with lower retention (~3.6 min, 17%) is detected on UV (lower), corresponding to partial oxidation and dimerization of C2Am prior to radiolabeling. This larger protein, however, is not radiolabeled. Note that the UV detector is installed upstream from the radio-detector on the HPLC system.
